# *Helicobacter pylori* Infection and Its Risk Factors: A Prospective Cross-Sectional Study in Resource-Limited Settings of Northwest Ethiopia

**DOI:** 10.1155/2018/9463710

**Published:** 2018-10-18

**Authors:** Markos Negash, Habtamu Wondifraw Baynes, Demeke Geremew

**Affiliations:** ^1^Department of Immunology and Molecular Biology, School of Biomedical and Laboratory Sciences, College of Medicine and Health Sciences, University of Gondar, Gondar, Ethiopia; ^2^Department of Clinical Chemistry, School of Biomedical and Laboratory Sciences, College of Medicine and Health Sciences, University of Gondar, Gondar, Ethiopia

## Abstract

**Background:**

*Helicobacter pylori (H. pylori) is* implicated for the causation of gastrointestinal tract infections including gastric cancer. Although the infection is prevalent globally, the impact is immense in countries with poor environmental and socioeconomic status including Ethiopia. Epidemiological study on the magnitude of *H. pylori* and possible risk factors has priceless implication. Therefore, in this study, we determined the prevalence and risk factors of *H. pylori* infection in the resource-limited area of northwest Ethiopia.

**Methods:**

A prospective cross-sectional study was conducted on northwest Ethiopia among 201 systematically selected dyspeptic patients. Data were collected using a structured and pretested questionnaire, and stool and serum samples were collected and analyzed by SD BIOLINE *H. pylori* Ag and dBest *H. pylori* Disk tests, respectively. Chi-square test was performed to see association between variables, and binary and multinomial regression tests were performed to identify potential risk factors. *P* values <0.05 were taken statistically significant.

**Result:**

Prevalence of *H. pylori* was found to be 71.1% (143/201) and 37.3% (75/201) using the dBest *H. pylori* Test Disk and SD BIOLINE *H. pylori* Ag test, respectively. *H. pylori* seropositivity, using dBest *H. pylori* Disk tests, is significantly associated in age groups <10 years (*P*=0.044) and married patients (*P*=0.016). In those patients with *H. pylori* (a positive result with either the Ab or Ag test), drinking water from well sources had 2.23 times risk of getting *H. pylori* infection (*P*=0.017), and drinking coffee (1.51 (0.79–2.96, *P*=0.025)) and chat chewing (1.78 (1.02–3.46, *P*=0.008) are the common risk factors.

**Conclusion:**

The present study discovered considerable magnitude of *H. pylori* among the dyspeptic patients in the study area*. H. pylori* infection is frequent in individuals drinking water from well sources, and thus, poor sanitation and unhygienic water supply are contributing factors. Policies aiming at improving the socioeconomic status will reduce potential sources of infection, transmission, and ultimately the prevalence and incidence of *H. pylori*.

## 1. Background

*Helicobacter pylori (H. pylori)* was the first formally recognized bacterial carcinogen. It has been etiologically associated with gastritis, peptic ulcer disease, gastric adenocarcinoma, and primary gastric lymphoma [1, 2].

*Helicobacter pylori* (*H. pylori*) colonizes 70–90% of the population in developing countries, whereas it is around 50% in developed countries [3–5]. In developing countries, an early childhood acquisition of *H. pylori* (30–50%) reaching over 90% during adulthood is the pattern of infection. Unless treated, colonization persists lifelong. *H. pylori* infection has been attributed to poor socioeconomic status, poor hygienic practice, and overcrowding condition [6, 7], a whole mark in developing countries.

The bacterium differs genetically, survives in harsh acidic gastric environment, and currently develops resistance for several antibiotics. Although epidemiological distribution of *H. pylori* varies globally, the magnitude of *H. pylori* has been shown to be 70.1% (Africa), 69.4% (South America), 66.6% (Western Asia), 34.3% (Western Europe), and 37.1% (North America) [8–10].

The prevalence of *H. pylori* in the Ethiopian dyspeptic patients is similarly high to other developing countries because most Ethiopian population live in households with low socioeconomic status and hygiene [7, 11, 12]. Magnitude of *H. pylori* among the outpatient department (based on a test kit detecting Immunoglobulin G (IgG) antibodies) at the University of Gondar Hospital (UOG Hospital) was ranged between 65.7% and 85.6% [13, 14]. Besides, it is a common reason to seek primary healthcare service and accounts for 10% of hospital admissions [15, 16].

All previous prevalence researches in the study area were conducted using IgG and/or IgM antibody rapid tests which have questionable performance in detecting acute infection and distinguishing active infection from previous exposure. Hence, the current study was conducted with an aim to determine the prevalence of *H. pylori* infection among the dyspeptic patients attending the UOG hospital in northwest Ethiopia, using stool antigen as well as serum antibodies technique and assessing potential risk factors.

## 2. Methods

### 2.1. Study Design, Period, and Area

This is a facility-based cross-sectional study which was conducted on patients with dyspepsia from February to March 2016 at the University of Gondar Hospital, Gondar, Ethiopia. The University of Gondar Hospital is one of the pioneer teaching hospitals in Ethiopia conducting community-based researches, providing teaching and diagnostic services for more than 5 million inhabitants.

### 2.2. Study Participants and Clinical Data Collection

After informed consent was taken from the dyspeptic patients, who visited the hospital outpatient department, suspected of *H. pylori* infection, all relevant clinical and sociodemographic data were collected using a structured and pretested questionnaire by trained data collectors.

### 2.3. Specimen Collection and Processing

Stool and blood specimens were collected from each patient for *H. pylori* antigen and antibody tests, respectively. The blood was centrifuged until serum is separated and stored in −20°C. The stool specimens were also stored in −20°C until the tests were performed. For this study, we followed the methods of Negash et al. [17] which has been evaluated four *H. pylori* diagnostic tests in the study area.

#### 2.3.1. SD Bioline *H. pylori* Ag Test (Standard Diagnostic, Inc., Korea)

Principle: the SD BIOLINE *H. pylori* Ag rapid test kit result window has 2 precoated lines, “T” (Test Line) and “C” (Control Line). Both the Test Line and the Control Line in the result window are not visible before applying any samples. The “T'” window coated with monoclonal anti-*H. pylori* will form a line after the addition of the stool specimen (if there is *H. pylori* antigen). The Control window is used for the procedural control, and a line should always appear if the test procedure is performed correctly, and the test reagents are working [17].

#### 2.3.2. dBest *H. pylori* Test Disk (Ameritech Diagnostic Reagent Co., Ltd., Tongxiang, Zhejiang, China)

Principle: this test contains a membrane strip, which is precoated with *H. pylori* capture antigen on the test band region. The *H. pylori* antigen-colloid gold conjugate and serum sample moves along the membrane chromatographically to the test region (T) and forms a visible line as the antigen-antibody-antigen gold particle complex forms. This test device has a letter of T and C as “Test Line” and “Control Line” on the surface of the case. Both the test line and control line in the result window are not visible before applying any samples. The control line is used for the procedural control. Control line should always appear if the test procedure is performed properly, and the test reagents of the control line are working [17].

### 2.4. Statistical Analysis

The data were cleaned and double entered on the excel spread sheet and transported to Statistical Package for Social Sciences (SPSS). The chi-square test was performed to see association between dependent and independent variables. Binary logistic regression and multinomial regression tests were performed to identify potential risk factors of *H. pylori* infection. *P* value less than 0.05 were considered statistically significant.

## 3. Result

### 3.1. Demographic Characteristics

A total of 201 dyspeptic patients were included in the study, and serum and stool samples were analyzed by dBest *H. pylori* Test Disk and SD BIOLINE *H. pylori* Ag tests, respectively. The mean ± SD (range) age of the participants was 29.5 ± 14.85 (7–85) years with a median of 23 years. The majority (140) of the study participants were male (69%), study subjects from the urban area (141) accounted 70%, and 69 (34.3%) of the participants were married. Of 201 participants, 104 (51%) were students, 38 (18.9%) were farmers, and 23 (11.4%) were house wives (Table 1). In this study, participants who were diagnosed as positive to the *H. pylori* stool antigen test were immediately commenced appropriate therapy.

### 3.2. Prevalence of *H. pylori* with respect to Sociodemography of Participants

Accordingly, the prevalence of *H. pylori* was found to be 71.1% (143/201) and 37.3% (75/201) using the dBest *H. pylori* Ab Test Disk (95% CI: 64.2–77.6) and SD BIOLINE *H. pylori* Ag test (95% CI: 30.3–44.3), respectively (Table 2). The highest prevalence of *H. pylori* infection was seen among the males than the females (98 vs 45 by Ab test and 79 vs 27 by Ag test), and *H. pylori* is more frequent in individuals living from the urban area than rural (101 vs 42 using the Ab test and 76 vs 30 using the Ag test), respectively. Regarding the occupational status, the students are the majority groups who come up positive for *H. pylori* (both in the Ab and Ag tests) than others, and meanwhile *H. pylori* seropositivity, using the dBest *H. pylori* Disk tests, is significantly associated with the age groups <10 years (*P* value = 0.044) and married patients (*P* value = 0.016) (Table 1).

### 3.3. *H. pylori* Infection across Clinical Parameters and Associated Risk Factors

Clinically, the patients with heartburn, abdominal fullness, and belching had come up with positive for the *H. pylori* tests, and likewise, belching is significantly associated (*P*=0.038), in logistic regression, with the antibody test. In those patients with *H. pylori* (a positive result with either a Ab or Ag test), drinking water from well sources had 2.23 times risk of getting *H. pylori* infection (*P*=0.017), and drinking coffee (1.51 (0.79–2.96, *P*=0.025) and chat chewing (1.78 (1.02–3.46, *P*=0.008) are the most common risk factors (Tables 3 and 4).

## 4. Discussion

A recent study demonstrated that 65.3% of the patients were positive for *H. pylori* IgG using the immunochromatographic method [13]. This shows that the current prevalence of *H. pylori* infection based on antibodies is much lower. The current 37.3% magnitude of *H. pylori*, using the SD BIOLINE *H. pylori* Ag test, is lower than a 52.3% and 53% of report from Ethiopia [18, 19] and studies from African and Asian countries [20–22]. The variation for these findings might be the difference in the socioeconomic factors, exposure for risk factors, study settings, and essentially the variability in the diagnostic methods.

The present study revealed that *H. pylori* seropositivity has been associated with age. In developing nations, where the majority of children are infected before the age of 10, the prevalence in adults peaks at more than 80% before age 50 [23–25]. While in developed countries, evidence of infection in children is unusual but becomes more common later on adulthood. In this study, the increment in serological positivity of *H. pylori* is seen starting from children through adulthood which reaches the peak on 18–30 age groups (68 (55.7%)), but cases are becoming lower as the age gets older and older. Within any age group, infection appears to be more common in blacks and Hispanics compared to the white population; these differences are probably in part related to socioeconomic factors [26, 27].

The increased prevalence of infection with age was initially thought to represent a continuing rate of bacterial acquisition throughout one's lifetime. However, epidemiologic evidence now indicates most infections are acquired during childhood even in developed countries [24, 28]. Most infections were acquired before five years of age with a declining incidence thereafter in one report from Ireland [29]. Thus, the frequency of *H. pylori* infection for any age group in any locality reflects that particular cohort's rate of bacterial acquisition during childhood years [28]. The organisms can be cultured from vomitus or diarrheal stools suggesting the potential for transmission among family members during periods of illness [30, 31].

The risk of acquiring *H. pylori* infection is related to the socioeconomic status and living conditions early in life. Factors such as density of housing, overcrowding, number of siblings, sharing a bed, and lack of running water have all been linked to a higher acquisition rate of *H. pylori* infection [32–34]. Our study proved that risk factors for acquiring *H. pylori* infection are most prevalent in the patients with *H. pylori* infection. Moreover, studies in the developing countries continue to show that childhood hygiene practices, and family education determines the prevalence of *H. pylori* infection [35]. In this study, illiterate individual accounts the majority (40/143 were positive for *H. pylori* Ab, and 24/106 were positive for *H. pylori* Ag tests) of *H. pylori* cases next to those who visited college. The association of *H. pylori* infection with the level of education, income, and race/ethnicity is not unique to *H. pylori,* since similar associations have been described with other chronic infections including cytomegalovirus, *herpes simplex virus-1*, and hepatitis B [36]. Studies indicated that declination of *H. pylori* infection has been attributed to economic progress and improvement in sanitation [37]. This study revealed that most (101/143 (antibody); 76/106 (antigen)) *H. pylori* positive cases are from the urban areas indicating that urbanization accompanied with poor sanitation.

The route by which infection occurs remains unknown, but multiple ways of transmission are reported [38, 39]. Person-to-person transmission of *H. pylori* through either fecal/oral or oral/oral seems most likely [31, 39]. Humans appear to be the major reservoir of infection; however, *H. pylorus has* been isolated from primates in captivity and from domestic cats [40, 41]. One report described the identification of *H. pylori* in milk and gastric tissue of sheep suggesting that sheep may be a natural host for the organism [42]. This may explain the higher infection rate that has been observed among shepherds compared to their siblings [43]. Similarly in our study, form the total *H. pylori* cases, farmers accounted the second highest proportion showing that close contact with domestic cattle may potentially result *H. pylori* transmission.

In addition to fecal/oral transmission of bacteria, contaminated water supplies in developing countries may serve as an environmental source of bacteria. In this study, majority (111/143 (antibody), 86/106 (antigen)) of *H. pylori* positive individuals use water sources from pipeline. The organism remains viable in water for several days and, using the polymerase chain reaction techniques, evidence of *H. pylori* can be found in most samples of municipal water from the endemic areas of infection [44–46]. Children who regularly swim in rivers, streams, and pools drink stream water, or eat uncooked vegetables are more likely to be infected [47]. *H. pylori* have been cultured from diarrheal stools of children in Gambia, West Africa, where almost all inhabitants are infected by five years of age [48].

Intrafamilial clustering of infection further supports person-to-person transmission. Infected individuals are more likely to have infected spouses and children than uninfected individuals [34, 49]. A study of children in Columbia found that the risk of infection correlated directly with the number of children aged 2 to 9 in the household, while younger children were more likely to be infected if older siblings were also infected [50]. Isolation of genetically identical strains of *H. pylori* from multiple family members [51] and custodial patients in the same institution [52] and further studies support transmission among persons sharing the same living environment. In addition to the familial type of transmission that occurs in developed and other nations, horizontal transmission between persons who do not belong to a core family also appears to take place in countries where the prevalence of infection is high [49]. As revealed by studies conducted on Ethiopia and Thailand [14, 53, 54], *H. pylori* infection is higher in married individuals demonstrating that cluster living environment has an impact on *H. pylori* transmission.

At last, it should be considered that the dyspeptic patients, other than the present serum antibody and stool antigen tests, did not undergo further confirmatory tests (endoscopy with biopsy for the histology culture and/or the very least urea breath test) due to economic constraints.

## 5. Conclusion

The present study discovered considerable magnitude of *H. pylori* in the study area*. H. pylori* infection is frequent in individuals drinking water from well sources, and thus, poor sanitation and unhygienic water supply are contributing factors. Policies aiming at improving the socioeconomic status will reduce potential sources of infection, transmission, and ultimately the prevalence and incidence of *H. pylori* infection.

## Figures and Tables

**Table 1:** Prevalence of *H. pylori* infection among the dyspeptic patients across sociodemographic characteristics at the University of Gondar Hospital Outpatient Department, *N*=201.

Sociodemographic characteristics		Positive for the Ab test, *N* (%)	Positive for the Ag test, *N* (%)	Total, *N* (%)
Sex	Male	98 (70)	79 (56.7)	140 (69.7)
	Female	45 (73.8)	27 (44.3)	61 (30.3)

Age (years)	<10	1 (50)^*∗*^	1 (50)	2 (1)
	10–19	10 (55.6)	12 (66.7)	18 (9)
	20–29	82 (67.2)	68 (55.7)	122 (60.6)
	30–39	12 (66.7)	6 (33.3)	18 (9)
	40–49	13 (92.9)	9 (64.3)	14 (7)
	50–59	12 (92.3)	5 (38.5)	13 (6.4)
	≥60	13 (92.9)	5 (35.7)	14 (7)

Residence	Urban	101 (71.6)	76 (53.9)	141 (70.1)
	Rural	42 (70)	30 (50)	60 (29.9)

Occupation	Farmer	30 (78.9)	19 (50)	38 (18.9)
	Student	73 (70.2)	64 (61.5)	104 (51.7)
	Government	14 (63.6)	8 (36.4)	22 (11)
	House wife	17 (73.9)	9 (39.1)	23 (11.4)
	Merchant	7 (87.5)	4 (50)	8 (4)
	No jobs	2 (33.3)	2 (33.3)	6 (3)

Education	Illiterate	40 (80)	24 (48)	50 (24.9)
	Primary	12 (63.2)	8 (42.1)	19 (9.5)
	Secondary	15 (62.5)	10 (41.7)	24 (11.9)
	College	76 (70.4)	64 (49.3)	108 (53.7)

Marital status	Married	56 (81.2)^*∗∗*^	34 (49.3)	69 (34.3)
	Single	87 (65.9)	72 (54.5)	132 (65.7)

Number of siblings	0	94 (67.1)	80 (57.1)	140 (69.7)
	1–4	29 (76.3)	15 (39.5)	38 (18.9)
	5–10	20 (87)	11 (47.8)	23 (11.4)

*N* = number; Ag = antigen; Ab = antibody. ^*∗*^*P* value = 0.044; ^*∗∗*^*P* value = 0.016.

**Table 2:** Prevalence of *H. pylori* infection among the dyspeptic patients attending the University of Gondar Hospital Outpatient Department, *N*=201.

Serologic tests	Prevalence of *H. pylori*			
	*N*	Percent (%)	SE	95% CI
dBest *H. pylori* Ab rapid test	143	71.1	3.2	64.2–77.6
SD BIOLINE *H. pylori* Ag test	75	37.3	3.5	30.3–44.3

*N* = number; SE = standard error; CI = confidence interval; Ag = antigen; Ab = Antibody.

**Table 3:** Prevalence of *H. pylori* infection among the dyspeptic patients across risk factors at the University of Gondar Hospital Outpatient Department, *N*=201.

Risk factors		Positive for the Ab test, *N* (%)	Positive for the Ag test, *N* (%)	Total, *N* (%)	Multivariate	*P* value
					OR (95% CI)	
Water source	Pipeline	111 (69.4)	86 (53.8)	160 (79.6)	2.23 (1.26–4.46)	0.017
	River	27 (77.1)	15 (42.9)	35 (17.4)		
	Well	5 (83.3)	5 (83.3)	6 (3)		
Washing hands with soap		87 (73.1)	63 (52.9)	119 (59.2)	1.04 (0.39–2.9)	0.743
Using toilet		69 (73.4)	49 (52.1)	94 (46.8)	1.80 (0.62–6.48)	0.496
Drinking alcohol		40 (65.6)	34 (55.7)	61 (30.3)	1.02 (0.48–2.9)	0.949
Drinking coffee		69 (74.2)	51 (54.8)	93 (46.3)	1.51 (0.79–2.96)	0.025
Chat chewing		4 (80)	4 (80)	5 (2.5)	1.78 (1.02–3.46)	0.008

**Table 4:** Prevalence of *H. pylori* infection among the dyspeptic patients across clinical parameters at the University of Gondar Hospital Outpatient Department, *N*=201.

Clinical parameters	Positive for the Ab test, *N* (%)	Positive for the Ag test, *N* (%)	Total, *N* (%)
Heartburn	139 (70.6)	104 (47.2)	197 (98)
Epigastric pain	139 (70.9)	103 (52.6)	196 (97.5)
Abdominal fullness	133 (70.4)	101 (53.4)	189 (94)
Vomiting	51 (72.9)	41 (58.6)	70 (34.8)
Nausea	106 (71.1)	83 (55.7)	149 (74.1)
Belching	110 (71.4)	75 (48.7)^*∗*^	154 (76.6)
Melena	43 (71.7)	27 (45)	60 (29.9)
Bloody vomiting	14 (87.5)	12 (75)	16 (8)

*N* = number; Ab = antibody; Ag = antigen. ^*∗*^*P* value = 0.038.

## Data Availability

The dataset supporting the conclusions of this article is included within the article.

## Abbreviations

Ab: Antibody
Ag: Antigen
IgG: Immunoglobulin G
IgM: Immunoglobulin M
IRB: Institutional Review Board
SD: Standard deviation
SPSS: Statistical Package for Social Sciences
UOG: University of Gondar Hospital.
